# *SMYD2* promoter DNA methylation is associated with abdominal aortic aneurysm (AAA) and *SMYD2* expression in vascular smooth muscle cells

**DOI:** 10.1186/s13148-018-0460-9

**Published:** 2018-03-02

**Authors:** Bradley J. Toghill, Athanasios Saratzis, Peter J. Freeman, Nicolas Sylvius, UKAGS collaborators, Rajiv Pathak, Marcus J. Brooks, Paul Hayes, Chris H. Imray, John Quarmby, Sohail A. Choksy, Jonothon J. Earnshaw, Cliff P. Shearman, Eric Grocott, Thomas Rix, Ian C. Chetter, William Tennant, Gabor Libertiny, Tim Sykes, Mark Dayer, Lynda Pike, Arun Pherwani, Colin Nice, Neil Browning, Charles N. McCollum, Syed W. Yusuf, Mark Gannon, Jamie Barwell, Sara Baker, Srinivasa R. Vallabhaneni, J. V. Smyth, Alun H. Davies, Tim Lees, Louis Fligelstone, Rob Sayers, Nilesh J. Samani, Mike J. Sweeting, John Thompson, Matthew J. Bown

**Affiliations:** 1 Department of Cardiovascular Sciences and the NIHR Leicester Biomedical Research Centre, University of Leicester, Leicester, LE2 7LX UK; 2 Department of Genetics and Genome Biology, University of Leicester, Leicester, LE1 7RH UK

**Keywords:** Epigenetics, Vascular disease, Aneurysm, Inflammation

## Abstract

**Background:**

Abdominal aortic aneurysm (AAA) is a deadly cardiovascular disease characterised by the gradual, irreversible dilation of the abdominal aorta. AAA is a complex genetic disease but little is known about the role of epigenetics. Our objective was to determine if global DNA methylation and CpG-specific methylation at known AAA risk loci is associated with AAA, and the functional effects of methylation changes.

**Results:**

We assessed global methylation in peripheral blood mononuclear cell DNA from 92 individuals with AAA and 93 controls using enzyme-linked immunosorbent assays, identifying hyper-methylation in those with large AAA and a positive linear association with AAA diameter (*P* < 0.0001, *R*^2^ = 0.3175).

We then determined CpG methylation status of regulatory regions in genes located at AAA risk loci identified in genome-wide association studies, using bisulphite next-generation sequencing (NGS) in vascular smooth muscle cells (VSMCs) taken from aortic tissues of 44 individuals (24 AAAs and 20 controls). In *IL6R*, 2 CpGs were hyper-methylated (*P* = 0.0145); in *ERG*, 13 CpGs were hyper-methylated (*P* = 0.0005); in *SERPINB9*, 6 CpGs were hypo-methylated (*P* = 0.0037) and 1 CpG was hyper-methylated (*P* = 0.0098); and in *SMYD2*, 4 CpGs were hypo-methylated (*P* = 0.0012).

RT-qPCR was performed for each differentially methylated gene on mRNA from the same VSMCs and compared with methylation. This analysis revealed downregulation of *SMYD2* and *SERPINB9* in AAA, and a direct linear relationship between *SMYD2* promoter methylation and *SMYD2* expression (*P* = 0.038). Furthermore, downregulation of *SMYD2* at the site of aneurysm in the aortic wall was further corroborated in 6 of the same samples used for methylation and gene expression analysis with immunohistochemistry.

**Conclusions:**

This study is the first to assess DNA methylation in VSMCs from individuals with AAA using NGS, and provides further evidence there is an epigenetic basis to AAA. Our study shows that methylation status of the *SMYD2* promoter may be linked with decreased *SMYD2* expression in disease pathobiology. In support of our work, downregulated *SMYD2* has previously been associated with adverse cardiovascular physiology and inflammation, which are both hallmarks of AAA. The identification of such adverse epigenetic modifications could potentially contribute towards the development of epigenetic treatment strategies in the future.

**Electronic supplementary material:**

The online version of this article (10.1186/s13148-018-0460-9) contains supplementary material, which is available to authorized users.

## Background

Abdominal aortic aneurysm (AAA) is a degenerative cardiovascular disease and a global health concern. AAA is responsible for between 2 and 4% of deaths in white males over the age of 65 [1–3]. Progressive aneurysm growth may lead to rupture, characterised by internal aortic haemorrhage, which results in death in approximately 80% of cases. Surgical repair is the only proven therapeutic option in order to prevent or treat AAA rupture. The exact etiological basis of AAA is still not fully understood, making it difficult to develop a successful pharmaco-therapeutic strategy [4]. However, it is well known that AAA is multifactorial with known risk factors that contribute towards aneurysm development (smoking, male sex, increased age, white European ancestry, atherosclerosis and hyperlipidaemia) [5, 6]. AAA also demonstrates strong heritability [7, 8] and is polygenic. To date, 10 genomic risk loci have been identified through the conduct of genome-wide association studies (GWASs) [9–16].

Epigenetics refers to modifications of the genome that are not exclusively a result of change in the primary DNA sequence and includes DNA methylation, histone modifications and non-coding RNAs [6, 17]. These mechanisms are essential for cell, tissue and organ development and directly interact with DNA sequence/structure to manipulate gene expression at the transcriptional and post-transcriptional levels. However, aberrant epigenetic modifications have been implicated in many complex diseases [18–20]. DNA methylation is the most extensively studied epigenetic modification to DNA and involves the addition of a methyl group to a cytosine base 5′ to a guanine (CpG dinucleotide) by DNA methyltransferase enzymes, which can inhibit gene transcription; however, increased methylation has also been associated with increased expression, since methylation can be necessary for transcriptional binding [21–23]. DNA methylation patterns are long-term inherited signatures that are passed through generations and can be affected by both genetic and environmental factors [24, 25].

There is currently very limited investigation surrounding the role of DNA methylation in AAA, and the only published study to date was conducted by Ryer et al. [26], who performed genome-wide methylation analysis in peripheral blood DNA from 20 AAA patients (11 smokers and 9 non-smokers) and 21 control samples (10 smokers and 11 non-smokers) using Illumina 450k micro-arrays. They identified differentially methylated regions in *ADCY10P1*, *CNN2*, *KLHL35* and *SERPINB9*, and differential expression in *SERPINB9* and *CNN2*.

Variations in DNA sequence at polymorphic loci can result in variable patterns of DNA methylation, known as methylation quantitative trait loci (meQTL) [27–29]. Disease variants can alter transcription factor levels and methylation of their binding sites [30]. The direct mechanisms of the variants associated with AAA are not fully known, but many are intronic and it is likely that their effects are regulatory in nature as opposed to being directly functional. It is therefore feasible that disease-specific meQTLs exist for AAA, and considering many meQTL act in cis [28], their likely effects on methylation are in the genes surrounding the risk loci identified in previous GWASs.

In this study, targeted bisulphite next-generation sequencing (NGS) was performed in vascular smooth muscle cell (VSMC) DNA, isolated from whole aortic tissues of AAA and controls, to investigate the methylation status of CpG islands in regulatory regions of genes located near AAA genomic risk loci. This approach offers a more refined way to assess the methylation status of genes in proximity to AAA risk loci than other methodologies such as the 450k microarray. The identification of adverse epigenetic modifications directly linked to patients with AAA could offer a more comprehensive understanding of AAA pathobiology, and an alternative research avenue in the search for a future treatment strategy [31, 32].

## Results

### Global DNA methylation and homocysteine analysis in AAA patients and controls

**Fig. 1:**
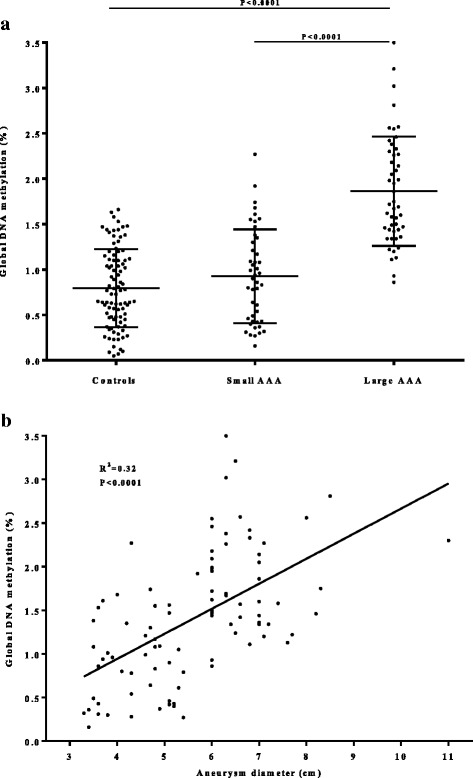
**a** Global DNA methylation levels of peripheral blood DNA in controls (aortic diameter < 25 mm, *n* = 92), small aneurysms (aneurysm diameter 30–55 mm, *n* = 45) and large aneurysms (aneurysm diameter > 55 mm, *n* = 48). **b** The linear relationship between AAA size and DNA methylation (*n* = 93)

Global genomic DNA methylation was assessed in peripheral blood DNA of 185 individuals, and circulating homocysteine (HCY) was assessed in blood plasma from 137 of the same individuals using ELISAs. Global DNA methylation was significantly higher in men with large AAA (> 55 mm, *n* = 48, global DNA methylation 1.86% (± 0.6%)) compared to men with small AAA (30–55 mm, *n* = 45, global DNA methylation 0.93% (± 0.52)) and controls (< 25 mm, *n* = 92, global DNA methylation 0.79% (± 0.43%)) (Fig. 1a). There was a linear relationship between AAA size and global DNA methylation which was adjusted for patient age (Fig. 1b) (Pearson coefficient *R*^2^ value = 0.3175, *P* < 0.0001). Circulating blood plasma HCY levels were slightly higher in men with AAA compared to controls (9.6 μmol/L ± 0.62, *n* = 70, vs 7.94 μmol/L ± 0.52, *n* = 67, *P* = 0.0433) (Additional file 1: Figure S1a). However, there was no association between global methylation and circulating HCY (*P* = 0.095) (Additional file 1: Figure S1b).

### Bisulphite NGS of vascular smooth muscle cell DNA in AAA patients and controls

**Fig. 2:**
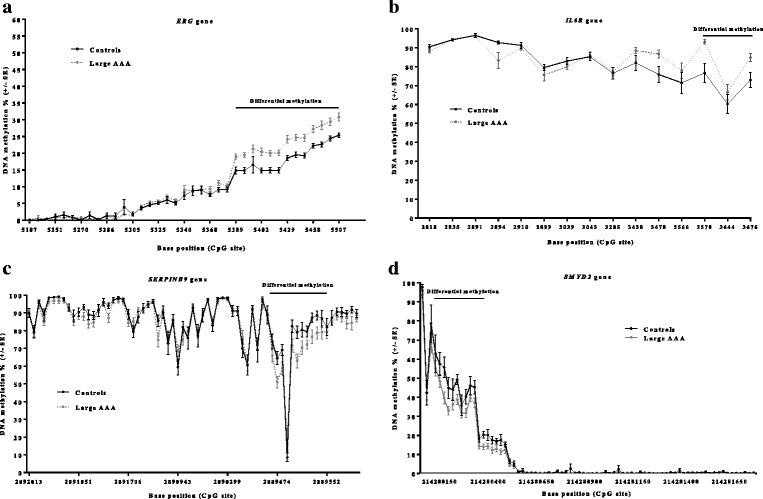
Vascular smooth muscle cell DNA methylation status of bisulphite-sequenced CpG islands where significant differential methylation was observed in 20 controls vs 24 AAAs (*ERG* (**a**), *IL6R* (**b**), *SERPINB9* (**c**) and *SMYD2* (**d**)). See Tables 2 and 3 for descriptive statistics of differentially methylated CpGs

We isolated CpG islands within regulatory regions of genes associated with AAA (*LRP1*, *ERG*, *MMP9*, *LDLR*, *IL6R*, *SORT1*, *SERPINB9*, *SMYD2* and *DAB2IP—*Table 1) in DNA extracted from the VSMCs of 20 controls and 24 large AAAs (Additional file 1: Table S2). Using targeted bisulphite NGS, we determined the methylation status of these regions and subsequently compared AAA to controls. We identified significant differences in methylation between AAA and controls in four genes: *ERG*, *IL6R*, *SERPINB9* and *SMYD2* (Fig. 2 and Table 2). There were no significant differences in methylation status between AAA and controls in *DAB2IP*, *LDLR*, *LRP1*, *MMP9*, *SORT1* and an alternative promoter in *ERG* (Additional file 1: Figure S2). A consistently high mapped sequencing depth was observed for each gene, and there were no significant differences in sequencing coverage between AAA and controls in any gene. Mean mapped sequencing coverage for AAA and controls for each gene is shown in Additional file 1: Figure S3.

**Table 1:** Candidate genes and amplicons for VSMC bisulphite NGS: Genomic locations, primer sequences, annealing temperatures and CpG coverage are displayed for each amplicon targeting candidate genes

Gene symbol	Chromosome and RefSeq ID	RefSeq ID coordinates	Primer sequence 5′-3’	Annealing temp (°C)	CpG coverage
*LRP1*		3875–4240	F: TAGAAGGGGGTAGTGATTAAAAGTA	54	13
			R: AAAACAAACCCTAATTTAAAAAAAA		
	Chr: 12	4274–4640	F: GGGTATTAAGGTGGGTTTTATTTT	57	25
	NG_016444.1		R: TACTCTAAAATTTCAAACTCCCTCC		
		4680–5036	F: GTATTAGGGAGGAGGGTTTAGTTAG	54	24
			R: TCCTCAATACATAAACCTAAAACTC		
*ERG*		282787–283319	F:TTTTATTAGGTAGTGGTTAGATTTAGTTTT	54	22
	Chr: 21		R: TACCCCCAATTAATAAATTCCAATATA		
	NG_029732.1	283331–283680	F: TTGGAATTTATTAATTGGGGGTATATAT	57	6
			R: ACACTATCTTTTACAAAATCAATCCAC		
		5162–5540	F: AAAATTTTTGGAAGGGGTTTAGTT	56	37
			R: ATAATATTTTTCCAACCTCATTAAAAAC		
*MMP-9*		3980–4579	F: TTGGGTTTAAGTAATTTTTTTATTT	52	7
	Chr: 20		R: TAACCCATCCTTAACCTTTTACAAC		
	NG_011468.1	4640–5600	F: GATGGGGGATTTTTTTAGTTTTATT	57	8
			R: TACCCATTTCTAACCATCACTACTC		
*LDLR*		4376–4725	F: TTTTTTAAGGGGAGAAATTAATATTTA	54	5
			R: CACAAAAAAATAACAACAACCTTTC		
		4776–5355	F: GAAAGGTTGTTGTTATTTTTTTGTG	57	28
	Chr: 19		R: AAACTCCCTCTCAACCTATTCTAAC		
	NG_009060.1	5464–5972	F: TTTAAGTTTTTATAGGGTGAGGGAT	56	31
			R: ACACCCAACTCAAAATAACAATAAC		
		6054–6785	F: AATTTTATTGGGTGTAGTTTAATAGGTTAT	54	49
			R: CTCAAAATCATACACTAACCAACCTC		
*IL6R*		2781–3061	F: TTTTTGTTTAGGTTGGAGTGTAGTG	52	8
	Chr: 1		R: CAAAATTTTAATATTATAATTCACATAAAA		
	NG_012087.1	3095–3729	F: GTTTGTTTTGGTGTAGAGATAGGTG	58	7
			R: ATAAAACTCCCAAATAAACAAAACC		
*SORT1*	Chr: 1	4363–4804	F: AGATTATTTTTTAGGTTTGTAGGAGTTA	55	24
	NG_028280.1		R: AACAAAAACTACTAAAATCAACCCC		
*SMYD2*		214280141–214280630	F: TTTTATTTTGAAGTAGTGGTTTTTGTTAA	55	13
			R: TTTTCCAAAATTAAAAATTTTTAAACCT		
	Chr: 1	214280631–214281330	F: AAGGTTTAAAAATTTTTAATTTTGGAAA	58	82
	NC_000001.11		R: AAATCCCCCACCTAAAAAAACTAC		
		214281331–214281733	F: TTTTTTTAGGTGGGGGATTT	55	46
			R: CCCACATTTAAAAACAAAAACCTAC		
*SERPINB9*		2891544–2892079	F: GGTGTTGAAAATATTTTTGGAGGTA	57	26
			R: AAAATCTACCATTCATCAAACTAACAAA		
		2890564–2891263	F: TTAGAGGGTGGGATTAGAGGTAGTT	54	9
	Chr: 6		R: TTTCCACCTAAAAAACCAAAATTAA		
	NC_000006.12	2889934–2890423	F: GTATTTGGGAATTGTTGATGTTTTT	55	11
			R: ACAACAAAAAATCATTATAATCTATTTTCT		
		2889425–2889933	F: AGAATTTTATATGTAATTTATTTTGG	54	31
			R: TATAATCCCAACACTTTAAAAAACC		
*DAB2IP*		121689899–121690502	F: TTGGAGTAGTTTGTTTGTTGTGTT	57	10
			R: TAAAAAATTAAATACCCAAAACCTC		
		121690573–121691062	F: TTGTTATATTTTAAGTTGAGATTTTGGG	57	6
	Chr: 9		R: TAACCACATAAAAACATTCCAAACA		
	NC_000009.12	121767430–121767872	F: GGAGAGGAGATAGGAAAGTTTTTAG	57	6
			R: CTAACCTTATCCCTTACAACCACTAC		
		121768273–121768692	F: AATGGAGTGGGAGTTTGTTATAGTG	57	11
			R: TAATAACAACTTTTAACCCCACCTC

**Table 2:** Mean observed CpG site methylation in vascular smooth muscle cell DNA after bisulphite sequencing of candidate genes (total *n* = 24 AAAs and 20 controls—Additional file 1: Table S2)

Gene, chromosome and RefSeq ID	RefSeq ID coordinate	Overall mean meth %	Overall mean meth %	Overall meth difference	*P* value	*Q* value	Age-adjusted
	(CpG site)	± SD (AAA *n* = 24)	± SD (controls *n* = 20)	(% ± SE)			odds ratio
ERG	5389	19.00 (3.48)	14.83 (4.42)	4.17 (1.23)	0.0019	0.0037	NS
Chr: 21	5393	19.43 (3.72)	14.83 (3.618)	4.60 (1.16)	0.0004	0.0010	NS
NG_029732.1	5400	21.26 (5.76)	16.50 (10.57)	4.76 (2.58)	0.0002	0.0010	6.70
	5402	20.43 (5.45)	14.83 (3.50)	5.60 (1.48)	0.0001	0.0009	NS
	5405	19.96 (4.15)	14.89 (3.43)	5.07 (1.21)	0.0001	0.0008	6.18
	5408	20.13 (4.20)	14.89 (3.51)	5.24 (1.23)	0.0001	0.0008	6.19
	5429	24.13 (5.26)	18.56 ((3.22)	5.58 (1.41)	0.0003	0.0010	NS
	5440	24.65 (4.49)	19.50 (3.40)	5.15 (1.28)	0.0001	0.0008	NS
	5442	24.57 (5.12)	19.28 (3.25)	5.29 (1.38)	0.0002	0.0009	NS
	5458	27.22 (4.88)	22.22 (3.15)	5.00 (1.33)	0.0003	0.0010	NS
	5468	28.35 (6.01)	22.67 (3.16)	5.68 (1.56)	0.0015	0.0032	NS
	5474	29.39 (5.55)	24.39 (3.48)	5.00 (1.50)	0.0012	0.0027	NS
	5507	30.83 (5.57)	25.33 (3.09)	5.49 (1.46)	0.0004	0.0010	NS
IL6R Chr: 1	3570	93.17 (5.62)	76.70 (23.02)	16.47 (4.85)	0.0113	0.0743	3.11
NG_012087.1	3676	84.92 (10.89)	73.00 (17.49)	11.92 (4.31)	0.0176	0.0773	NS
SERPINB9	2889674	51.00 (16.19)	64.47 (16.19)	−13.47 (4.97)	0.0063	0.0375	NS
Chr: 6	2889644	20.46 (20.29)	8.579 (10.64)	11.88 (5.14)	0.0098	0.0478	3.29
NC_000006.12	2889627	71.67 (18.37)	82.53 (14.94)	−10.86 (5.20)	0.0058	0.0375	NS
	2889620	62.88 (19.54)	79.11 (15.15)	−16.23 (5.47)	0.0017	0.0273	NS
	2889612	70.33 (19.08)	80.37 (17.96)	−10.04 (5.71)	0.0064	0.0375	NS
	2889568	76.58 (19.21)	87.21 (11.26)	−10.63 (4.97)	0.0012	0.0273	0.33
	2889564	78.67 16.34)	88.58 (10.65)	−9.91 (4.35)	0.0010	0.0273	NS
SMYD2	214280412	39.50 (14.74)	55.67 (16.24)	−16.17 (6.00)	0.0004	0.0200	NS
Chr: 1	214280441	32.91 (10.53)	44.80 (17.29)	−11.89 (4.87)	0.0014	0.0299	0.09
NC_000001.11	214280507	38.78 (12.67)	49.22 (7.93)	−10.44 (4.56)	0.0016	0.0299	0.06
	214280600	37.04 (11.02)	45.30 (11.43)	−8.26 (4.22)	0.0014	0.0299	0.03

*NS* not significant

The greatest differences in methylation were seen in the *ERG* gene, where 13 CpGs were consecutively hyper-methylated (Fig. 2a) in AAAs compared to controls. The mean methylation of these sites was 18.67% ± 3.92 in controls versus 23.8% ± 4.11 in cases with a mean increase in methylation of 5.13% ± 0.43 (*P* = 0.0005, *Q =* 0.0014). The 13 CpGs in *ERG* span a region of 118 bp (NG_029732.1:g.5389_5507|gom) which is located within an intronic region directly downstream from the first *ERG* exon.

We observed a more conservative site of potential differential methylation in the *IL6R* gene promoter upstream of the transcriptional start site, where we identified significant hyper-methylation of 2 CpGs (NG_012087.1:g.3570_3676|gom) in AAA (89% ± 5.83) vs controls (74.85% ± 2.62) (Fig. 2b). The mean increase in methylation was 14.2% ± 3.22 (*P* = 0.0145, *Q =* 0.076).

In the *SERPINB9* gene, seven CpGs located in the 3′ untranslated region downstream of exon 7 were found differentially methylated in AAA compared to controls. Overall, hypo-methylation of the region was observed (NC_000006.12:g.2889674_2889564|lom) where six CpGs were hypo-methylated (*P* = 0.0037, *Q =* 0.032) and one CpG was hyper-methylated (*P* = 0.0098, *Q =* 0.0478) (NC_000006.12:g.2889644|gom) (Fig. 2c). This is in contrast to the overall AAA hyper-methylation reported by Ryer et al., [26], which was conducted on peripheral blood DNA.

**Fig. 3:**
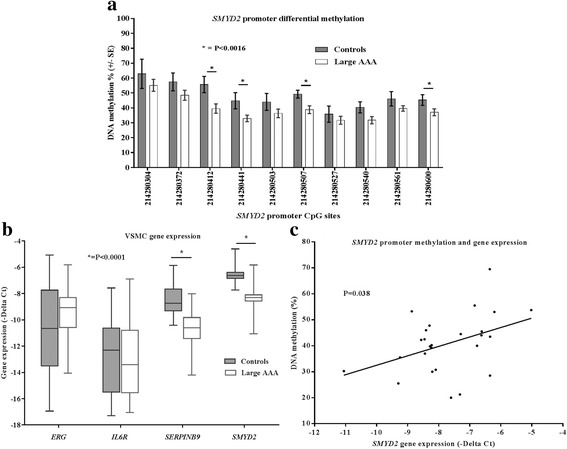
**a** Differentially methylated CpGs in the *SMYD2* gene promoter identified after bisulphite sequencing of DNA from 24 AAAs and 20 controls. **b** Relative gene expression levels of differentially methylated candidate genes (*SMYD2*, *SERPINB9*, *IL6R* and *ERG*) in vascular smooth muscle cells from 20 controls and 24 AAAs. **c** Linear relationship between gene expression and mean DNA methylation status of the differentially methylated CpGs (NC_000001.11: 214280412, 214280441, 214280507 and 214280600) in *SMYD2* (*n* = 26 where sufficient sequencing coverage and expression data was acquired)

Finally in the *SMYD2* gene, we identified four significantly hypo-methylated CpG sites in AAA compared to controls (Fig. 2d) in the gene promoter upstream of the transcriptional start site (NC_000001.11:g 214280412_214280600|lom). Mean control methylation was 48.75% ± 5.02, mean methylation in the cases was 37.06% ± 2.95, and the mean overall decrease in methylation in AAA compared to controls was 11.69% ± 8.2 (*P* = 0.0012, *Q =* 0.027). Figure 3a displays a more detailed, CpG-specific illustration of the differentially methylated sites in the *SMYD2* gene promoter.

Logistic regression was conducted on statistically significant differentially methylated CpGs in each gene to assess the potential effects of age (controls (56 years ±35) and AAA (68 years ±25)) on disease status. Some sites lost significance when applying age as an additional variable for differential methylation; however, there still remained CpGs in each differentially methylated gene that were significant independently of age. In addition, the effects of smoking and sex were assessed in our data by excluding all non-smokers and females respectively (Table 3).

**Table 3:** Mean observed CpG site methylation in vascular smooth muscle cell DNA after bisulphite sequencing of candidate genes after consideration of sex and smoking status as co-variates of methylation (total *n* = 24 AAAs, 16 male controls and 16 smoking controls—Additional file 1: Table S2)

Gene, chromosome and RefSeq ID	RefSeq ID coordinate	Meth % ± SD—all male smokers	Smokers only meth % ± SD	Smokers only meth difference (% ± SE)	Men only meth % ± SD	Men only meth difference (% ± SE)	Smokers only *P* value	Men only *P* value
	(CpG site)	(AAA—*n* = 24)	(Controls—*n* = 16)	*n* = 16	(Controls—*n* = 16)	*n* = 16	(24 AAAs vs 16 controls)	(24 AAAs vs 16 controls)
ERG	5389	19.00 (3.48)	14.43 (3.99)	4.57 (1.25)	15.07 (4.65)	3.93 (1.32)	0.0011	0.0059
Chr: 21	5393	19.43 (3.72)	14.86 (3.65)	4.58 (1.25)	14.80 (3.82)	4.63 (1.25)	0.0013	0.0009
NG_029732.1	5400	21.26 (5.76)	17.36 (11.73)	3.90 (2.88)	16.87 (11.58)	4.39 (2.82)	0.0011	0.0007
	5402	20.43 (5.45)	15.14 (3.55)	5.29 (1.64)	14.80 (3.73)	5.63 (1.61)	0.0009	0.0004
	5405	19.96 (4.15)	15.50 (3.54)	4.46 (1.33)	14.93 (3.65)	5.02 (1.32)	0.0014	0.0003
	5408	20.13 (4.20)	15.36 (3.69)	4.77 (1.36)	14.87 (11.57)	5.26 (1.33)	0.0007	0.0002
	5429	24.13 (5.26)	19.21 (2.83)	4.92 (1.53)	18.73 (3.26)	5.40 (1.52)	0.0028	0.0010
	5440	24.65 (4.49)	20.07 (2.92)	4.58 (1.35)	19.60 (3.48)	5.05 (1.37)	0.0010	0.0003
	5442	24.57 (5.12)	19.86 (2.51)	4.71 (1.47)	19.40 (3.36)	5.17 (1.50)	0.0009	0.0006
	5458	27.22 (4.88)	22.93 (2.49)	4.29 (1.41)	22.40 (3.31)	4.82 (1.44)	0.0030	0.0010
	5468	28.35 (6.01)	23.50 (2.34)	4.85 (1.68)	22.80 (3.39)	5.55 (1.71)	0.0127	0.0045
	5474	29.39 (5.55)	25.36 (2.56)	4.03 (1.58)	24.53 (3.70)	4.86 (1.63)	0.0125	0.0037
	5507	30.83 (5.57)	26.21 (1.85)	4.61 (1.54)	25.20 (3.36)	5.63 (1.60)	0.0040	0.0008
IL6R Chr: 1	3570	93.17 (5.62)	78.31 (22.41)	14.85 (4.76)	75.37 (23.77)	17.79 (5.02)	NS	0.0037
NG_012087.1	3676	84.92 (10.89)	75.56 (16.55)	9.35 (4.33)	73.63 (16.97)	11.29 (4.39)	NS	NS
SERPINB9	2889674	51.00 (16.19)	66.27 (15.98)	−15.27 (5.30)	64.07 (16.84)	−13.07 (5.41)	0.0037	0.0120
Chr: 6	2889644	20.46 (20.29)	8.13 (9.93)	12.33 (5.63)	9.27 (11.59)	11.19 (5.76)	0.0139	0.0230
NC_000006.12	2889627	71.67 (18.37)	84.93 (12.93)	−13.27 (5.44)	82.13 (15.91)	−10.47 (5.76)	0.0015	0.0100
	2889620	62.88 (19.54)	80.00 (15.17)	−17.13 (5.95)	81.00 (14.06)	−18.13 (5.84)	0.0018	0.0007
	2889612	70.33 (19.08)	80.27 (20.23)	−9.933 (6.43)	78.93 (19.58)	−8.60 (6.34)	0.0081	0.0168
	2889568	76.58 (19.21)	88.33 (12.19)	−11.75 (5.56)	87.67 (9.31)	−11.08 (5.33)	0.0003	0.0018
	2889564	78.67 16.34)	87.80 (11.82)	−9.13 (4.88)	90.47 (8.57)	−11.80 (4.59)	0.0039	0.0002
SMYD2	214280412	39.50 (14.74)	59.00 (17.12)	−19.50 (6.64)	55.38 (17.33)	−15.88 (6.37)	0.0002	0.0015
Chr: 1	214280441	32.91 (10.53)	48.57 (17.80)	−15.66 (5.37)	41.56 (14.78)	−8.64 (4.64)	0.0004	NS
NC_000001.11	214280507	38.78 (12.67)	50.14 (8.61)	−11.36 (5.14)	50.13 (7.97)	−11.34 (4.81)	0.0012	0.0009
	214280600	37.04 (11.02)	47.57 (10.63)	−10.53 (4.72)	45.78 (12.02)	−8.73 (4.44)	0.0001	0.0020

*NS* not significant

### Functional corroboration of differential methylation in AAA patients and controls

#### Gene expression analysis of differentially methylated genes

In genes where differentially methylated CpGs were identified between AAA and controls (*ERG*, *IL6R*, *SERPINB9* and *SMYD2)*, we performed mRNA expression analysis to assess the relationship between DNA methylation and gene expression. We identified a significant overall reduction in the expression of *SMYD2* (*P* < 0.0001) and *SERPINB9* mRNA (*P* < 0.0001) in people with AAA compared to controls. However, there were no differences in expression in the *IL6R* and *ERG* mRNA (Fig. 3b). Subsequently, the methylation values of significantly associated CpG regions were correlated with expression levels in their respective genes using linear regression. In the *SERPINB9* gene, where significant expression and methylation differences were observed, neither the hypo- nor hyper-methylated CpG sites were associated with *SERPINB9* expression levels (*P* = 0.243 and *P* = 0.31 respectively). Finally, the mean DNA methylation percentage of differentially methylated CpGs (214280412, 214280441, 214280507 and 214280600) in the *SMYD2* gene promoter was significantly associated with *SMYD2* gene expression (*P* = 0.0383, *R*^2^ = 0.17, (Fig. 3c). However *SMYD2* gene expression did not correlate with aortic diameter.

### Immunohistochemistry to assess Smyd2 in aortic tissues

To further corroborate the *SMYD2* methylation/expression relationship and to ensure that the differential gene activity seen between AAA and controls was applicable to the site of aneurysm in the aortic wall, histological staining of the Smyd2 protein was conducted in whole aortic tissues from six of the same samples used in methylation and gene expression analysis (three AAAs and three controls).

**Fig. 4:**
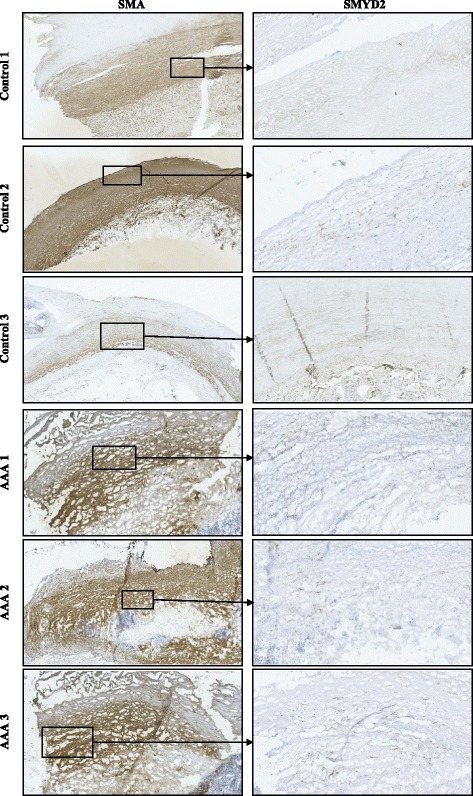
Immuno-histochemical staining: staining of aneurysmal (*n* = 3) and non-aneurysmal (*n* = 3) abdominal aortic frozen tissue sections for smooth muscle actin (SMA) and Smyd2. Brown = presence of primary specific antibody

For each of the six samples, three individual stains were conducted (total = 18 slides) on frozen tissue cuts from the same aortic sections. The first was for Smyd2 staining, the second was a negative Smyd2 control with no primary antibody, and the last was for smooth muscle actin (SMA) staining. The controls were checked for the presence of Smyd2, and all were absent of brown colouring, which represents the presence of primary antibody. The SMA slides were then visualised, and where the densest section of smooth muscle fibres was seen, the Smyd2 slides were visualised in the same regions, where photographs were taken (Fig. 4).

The histological technique used for this assay is hard to quantify and is not as objective as other methodologies. However, Fig. 4 subjectively shows a reduced abundance of the Smyd2 protein (brown colouring) in the tissues of those with AAA compared to controls. Whilst a statistical analysis is not available, it appears that the same pattern of downregulated Smyd2 activity (from the mRNA gene expression analysis) is evident in AAA compared to controls. This demonstrates that transcript expression differences are also reflected from a translational perspective at the site of aneurysm, which is in turn associated with *SMYD2* promoter methylation.

## Discussion

Peripheral blood global DNA hyper-methylation is commonly a hallmark of chronic inflammation and has been observed as a potential pathological marker of disease [19, 33–37], which supports our findings. In turn, inflammation is a pathological hallmark of AAA, yet up until now, no work has investigated global methylation in AAA. Our study has identified global DNA hyper-methylation in patients with a large AAA, and a clear linear relationship with AAA diameter in peripheral blood DNA. These results were adjusted for age, and both groups (cases and controls) consisted exclusively of white males with at least a 10-year smoking history. Age, smoking and inflammation, as stated, can have considerable impact upon global and Cp-specific DNA methylation patterns, consisting of global hypo-methylation (ageing), hyper- and hypo-methylation (smoking) and global hyper-methylation (inflammation). Each of these factors is also significant a risk factor for AAA [6].

In addition, circulating HCY has previously been associated with global methylation [38], is a proposed biomarker for cardiovascular disease [39] and has also been suggested to have a role in AAA [40]. However, the differences in HCY between AAA and controls in this study were not clinically different (hyper-homocysteinemia is described above 15 μmol/L) and support the idea that HCY is not an appropriate biomarker for AAA, which is also suggested by Lindqvist et al., [41]. In addition, there was no significant relationship between HCY and global DNA methylation, suggesting that global DNA hyper-methylation is associated with AAA independently of circulating blood HCY. Similarly to other inflammatory diseases, increased global DNA methylation may be a factor in the pathobiology of AAA and provides a solid rationale for further study in to the role of DNA methylation in AAA.

This study is the first to adopt bisulphite NGS, a gold standard technique in studies of DNA methylation [42], to identify differentially methylated CpG sites associated with AAA in VSMCs. VSMCs are the main constituents of the tunica media, where cellular apoptosis occurs as a hallmark of AAA during aneurysm development and growth [43]. Ryer et al. [26] previously conducted a methylation study on AAA in peripheral blood DNA using Illumina 450k micro-arrays and found differential methylation and gene expression in *CNN2* and *SERPINB9.* In VSMCs, we also identified differential methylation in *SERPINB9*, but the relationship was converse to that seen in DNA isolated from peripheral blood. In our study, after assessing the association between differential methylation and expression in *SERPINB9* with linear regression analysis, it was determined that there was not a direct relationship between the two. *SERPINB9* could however still be important in AAA, as reduced *SERPINB9* expression is associated with atherosclerotic disease progression and is inversely related to the extent of apoptosis within the intima [44].

Most importantly in this study, we identified significant DNA hypo-methylation in the *SYMD2* gene promoter in those with AAA (NC_000001.11:g214280412_214280600|lom), and the mean methylation percentage of the differentially methylated CpGs were significantly correlated with gene expression. *SMYD2* gene expression however was not correlated with aortic diameter, indicating that downregulated *SMYD2* expression, a potential result of *SMYD2* promoter methylation, may be associated with the development, and not progression, of AAA. Our analysis is limited by the fact that all VSMC samples in this study were from individuals with AAA greater than 55 mm. The lack of data from those with smaller AAA limits our ability to make conclusions on whether the level of *SMYD2* expression is linked to AAA size. Future studies should therefore address this. Further to this analysis, the decrease in expression of *SMYD2* in AAA was corroborated in aortic tissues using immunohistochemistry. *SMYD2* codes for an important lysine methyltransferase and is well known for its role in transcriptional regulation [45]. Our results are particularly relevant when considering the role of Smyd2 in inflammation and cardio-physiology, as previous studies have identified a role in cardio-protection and suppression of inflammation. In one study, protein levels of Smyd2 were decreased in cardiomyocytes after cellular apoptosis and after myocardial infarction. In addition, *SMYD2* deletion in cardiomyocytes in vivo promoted apoptotic cell death upon myocardial infarction [46]. It has also been reported that Smyd2 is a negative regulator for macrophage activation. Elevated *SMYD2* expression suppresses the production of pro-inflammatory cytokines, including IL-6 and TNF. In addition, macrophages with high *SMYD2* expression promote regulatory T cell differentiation as a result of increased TGF-β production and decreased IL-6 secretion [47]. In summary, previous work has demonstrated that down-regulation of *SMYD2* is linked with adverse cardio-physiology and an increase in inflammation, both of which are key hallmarks of AAA pathobiology.

Looking more closely at the relationship between *SMYD2* expression and DNA methylation in our study, it is clear that the observed association is opposite to the traditional notion of promoter hyper-methylation inhibiting gene transcription. However, it is becoming better known that this is not definitively correct, as multiple pieces of evidence go against this paradigm [48, 49]. In some cases, methylation is required for activation of transcription and is therefore positively correlated with gene expression [49], which could be appropriate to our findings. Due to this, it must be stated that the exact mechanisms by which methylation controls gene expression is still not definitively understood and is not always the same.

The results presented in this article are preliminary. Future work will concentrate on proving up the *SMYD2* relationship with large-scale replication, whilst also establishing whether there is a causal genetic basis to differential methylation patterns in AAA (meQTL analysis). There is some knowledge of meQTLs, but this is still very scarce and currently there is no comprehensive database highlighting phenotype-specific associations between genotype and methylation. It is therefore likely that existing disease-specific meQTLs are unreported, and in our case, genes that are known AAA risk loci represent potentially insightful targets for further genetic and methylation analysis. Finally, after replication, it is suggested that mechanistic analysis to assess the causal role of *SMYD2* promoter methylation on gene function would be insightful.

There were limitations to this work, including limited sample size which was hindered due to the difficulty of obtaining aortic tissues from individuals. This problem in turn had an effect on sample group demographics, and we could not control for gender, smoking and age as effectively as we would have liked—as we did in the peripheral blood global methylation assay.

## Conclusion

This pilot study is the first to assess DNA methylation in VSMCs of people with AAA, and the first to assess methylation in AAA using bisulphite NGS. The results presented in this article demonstrate further evidence that there is an epigenetic basis to AAA and is proof of concept for further large-scale analysis. Global peripheral blood DNA hyper-methylation is associated with large AAA, increasing AAA size, and this relationship appeared independently of circulating HCY. Gene-specific changes in DNA methylation were also identified in VSMC DNA (*IL6R*, *SMYD2*, *SERPINB9* and *ERG*). In particular, there was a significant association between the methylation status of CpGs in the *SMYD2* promoter and *SMYD2* gene expression, which was further corroborated in whole aortic tissues using immunohistochemistry. In summary, this article demonstrates that global and gene-specific DNA methylation changes exist in AAA and are potentially involved in the pathobiology of the disease and could be useful in the future development of epigenetic therapies.

## Methods

### Population

Patients and controls were recruited from two sources: The UK aneurysm growth study (UKAGS) (http://www2.le.ac.uk/projects/ukags) and our local AAA research programme based at the NIHR Leicester Cardiovascular Biomedical Research Centre. The UKAGS collects peripheral blood samples from men with AAA (aortic diameter 30 mm or greater) and healthy controls (all screened for AAA) recruited from the English NHS AAA Screening Programme. Furthermore, AAA biopsies (aortic wall) are taken from patients undergoing open AAA repair in our regional vascular unit, where we have also established an aortic tissue collection from cadaveric organ donors. Ethical approval by an NHS Research Ethics Committee was obtained for both studies. Risk factors associated with AAA can be confounders of DNA methylation and include age, smoking, gender and ethnicity. Where possible, to help prevent any confounding effects of these factors, white males over the age of 65 with at least a 10-year history of smoking were selected for inclusion.

### Vascular smooth muscle cell culture from aortic biopsies

Aortic tissues were collected by vascular surgeons within the vascular surgery group at the University of Leicester. There were no signs of AAA or atherosclerosis in the abdominal aortas obtained from organ donors, which were therefore used as controls. For each aortic tissue sample collected from those undergoing open AAA surgery (*n* = 24) and from cadaveric organ donors (*n* = 20), explant culture was performed to isolate and grow VSMCs in vitro. It was important to isolate individual cells given that independent cell lines have different epigenetic profiles. VSMCs are a good surrogate for such analysis considering aneurysmal formation is characterised by inflammation and VSMC apoptosis in the tunica media of the aortic wall.

Thin sections of the tunica media were carved from the whole aortic tissues no longer than 48 h after surgery (AAA samples), or death (cadaveric organ donor samples), and placed in T80 cell culture flasks containing 10 ml smooth muscle cell Medium 231 (ThermoFisher Scientific - M2315005) with added Smooth Muscle Growth Supplement (ThermoFisher Scientific - S00725). A 25-ml bottle of growth serum was added to each 500 ml 231 media prior to use. The flasks containing the media, growth supplement and aortic tissues were left to incubate at 37 °C until visible primary cell growth was observed (~ 2 weeks). After primary growth of VSMCs, confluent cells were detached from the flask with the addition of 2× trypsin-EDTA solution and incubated at 37 °C for 3 min. Five-millilitre sterile phosphate buffered saline (PBS) and fetal bovine serum solution (20:1 ratio) was added to neutralise the trypsin. Solutions containing detached cells were aspirated from the flasks into sterile universal tubes and centrifuged at 500×*g* for 6 min at 20 °C. Supernatants were discarded and cell pellets were re-suspended in 3 ml media. One millilitre of the 3 ml suspensions were added to three new T80 cell culture flasks, each containing 10 ml of media. This process was repeated until sufficient amounts of isolated VSMCs (two clearly visible cell pellets) were available for DNA and RNA extraction, however, never beyond passage 3 to attenuate any potential changes in cellular gene expression or DNA methylation patterns because of the artificial media/culture environment. Isolated cultured cells stored by cryogenic freezing at − 80 °C until needed for experimental analysis. In the earliest stages of establishing our explant culture work flows, α-smooth muscle immunofluorescent cell type staining (internal work not published) was conducted to assess for fibroblast contamination in our standard protocol, and this protocol was used in this study.

### DNA extraction

DNA was extracted from 185 peripheral blood mononuclear cell (PBMC) samples from the UKAGS for global methylation analysis (Additional file 1: Table S1), and 44 VSMC samples (Additional file 1: Table S2) isolated from whole aortic tissues for bisulphite NGS analysis using the DNeasy Blood & Tissue Kit (Qiagen) and according to the manufacturer’s standard protocol. DNA concentrations were measured using a NanoDrop Spectrophotometer. Each sample was diluted to 25 ng/μl using sterilised distilled water in a 100 μl final volume working solution.

### Global methylation analysis in peripheral blood DNA

Genome-wide global DNA methylation was assessed in DNA derived from the 185 PBMC samples from our UKAGS resource (Additional file 1: Table S1) using a colorimetric enzyme linked immunosorbent-assay (ELISA) following the manufacturers’ protocol (Epigentek - Methyl flash DNA quantification (colorimetric)). All reactions were performed in duplicate, and for each new 96-well plate, new standardisation controls were conducted. Mixed plate repeats were also performed to assess for potential batch effects. Absorbance (optical density (OD)) was measured with a micro-plate colorimetric spectrophotometer (Bio-Tek ELX808IU Ultra Microplate Reader), and ODs were converted to genome methylation percentage with the use of positive (5 ng of 50% methylated DNA) and negative control (20 ng of un-methylated DNA) reactions, which were also conducted in duplicate.

### Homocysteine analysis in circulating blood plasma

A total of 137 of the blood plasma samples from the global methylation assay were available (67 controls and 70 AAAs) for homocysteine (HCY) analysis (Additional file 1: Table S3). Colorimetric ELISAs were performed following the manufacturers’ protocol (Cell Biolabs Inc. – Homocysteine ELISA kit – STA-670). All reactions were conducted in duplicate with new controls and mixed plate repeats for each new 96-well ELISA plate. ODs were measured with a micro-plate colorimetric spectrophotometer and HCY levels were determined using the standard curve method.

### Bisulphite conversion of DNA and targeted Illumina NGS

Each of the 44 VSMC DNA samples isolated from aortic biopsies was bisulphite-converted using the MOD50 kit (Sigma–Aldrich) according to the manufacturer’s standard protocol. Eluted bisulphite-treated DNA was concentrated at 25 ng/μl in sterilised distilled water.

Genes for this study were chosen based on the results from the recent AAA GWAS meta-analysis conducted by Jones et al., [15], and included the following: low-density lipoprotein receptor-related protein 1 (*LRP1*), ETS-related gene (*ERG*), matrix metallopeptidase 9 (*MMP9*), low-density lipoprotein receptor (*LDLR*), interleukin 6 receptor *(IL6R*), sortilin 1, (*SORT1*), SET and MYND domain containing 2 (*SMYD2*) and DAB2-interacting protein (*DAB2IP*). In addition, *SERPINB9* was included, which was differentially methylated in PBMCs from patients with AAA in the Ryer et al. study previously described. We also tried to target the differentially methylated CpG Island in the *CNN2* gene from the Ryer et al. study but bisulphite-specific PCR primers could not be designed due to the repetitive nature of the target sequence.

Within all candidate genes (with the exception of the *SERPINB9* CpG Island which is located in the gene body), promoters and transcriptional start sites were identified using the transcriptional regulatory element promoter database (http://rulai.cshl.edu/TRED) and the NCBI gene database (https://www.ncbi.nlm.nih.gov/gene). All candidate gene sequences (GRCh38) were compiled in a single file for use as an in-house reference sequence for bioinformatics analysis.

Bisulphite-specific PCR primers were designed to isolate genomic regions of interest within candidate genes using methprimer [50] (Table 1). Each 20 μl bisulphite-specific PCR reaction consisted of 8 μl sterilised distilled water, 10 μl 2× jumpstart red-taq polymerase ready mix (SIGMA), 1 μl mixed forward and reverse primers (5 μM, SIGMA) and 1 μl bisulphite converted DNA (25 ng/μl). Each bisulphite-specific primer pair was optimised via PCR:

- 94 °C for 2 min—1 cycle
- 94 °C for 15 s—40 cycles
- 52 °C - 58 °C for 35 s—40 cycles
- 72 °C for 35 s—40 cycles
- 72 °C for 5 min—1 cycle

Amplicons were checked by fractionation using agarose gel electrophoresis (10 μl per well, 2% agarose gel).

All bisulphite-specific PCR reactions (total *n* = 1146) were cleaned with ExoSAP-IT PCR Clean-up (Affymetrix). On ice, 5 μl of the PCR reactions were mixed with 2 μl ExoSAP-IT solution and incubated at 37 °C for 15 min, then at 80 °C for 15 min. The cleaned products were pooled together (2 μl of each reaction) to create a single pooling of each separate DNA sample (44 individual samples containing 26 amplified genomic regions spanning 9 genes).

The NEBNext Ultra™ DNA Library Prep Kit for Illumina (E7370) and NEBNext Mutiplex Oligos for Illumina dual index kit (E7600) were used for end repair and adaptor ligation and quantification (bar coding for multiplex sequencing) using the standard manufacturer protocols. Clean-up of adaptor-ligated DNA was performed using Agencourt AMPure XP - PCR Purification (A63880) following the manufacturer’s standard protocol.

Each of the adaptor ligated, bar-coded DNA samples was ran on an Agilent 2100 Bioanalyzer Instrument (expert High-Sensitivity DNA chip) to assess sample DNA concentration and the presence and distribution of correct fragment sizes [51]. These readings were used to pool the individual samples prior to sequencing at 4 nM. Indexed libraries were pooled, and paired-end multiplexed sequencing (2 × 310 bp) was performed on an Illumina Miseq platform using MiSeq Reagent Kit v3. Sequencing was conducted in the NUCLEUS Genomics Centre, University of Leicester.

### Bioinformatics workflow and data analysis

A provisional data quality filter (Phred quality score (*Q*) = 15) was applied to fastq files with Trimmomatic [52] to remove all low-quality raw sequencing reads, remove contaminating Illumina adaptor sequences, and to clip and discard partially poor-quality sequencing reads four bases at a time using the sliding window function.

BWA-meth (https://github.com/brentp/bwa-meth) was used to align the provisionally filtered sequencing reads to the in house reference sequence. BWA-meth is specifically designed for targeted bisulphite NGS analysis.

PCR duplicates for all BAM files were marked and excluded with the Picard utility MarkDuplicates (http://broadinstitute.github.io/picard/>), and the final processed files were sorted and indexed with Samtools [53].

Finally, for each sample, the methylation values (normalised as methylation %) of each sequenced CpG in each individual were extracted to a bedGraph file at a threshold of Q50 (read alignment filter) and Q20 (base quality filter) with a minimum read depth of 5 using PileOMeth (https://github.com/dpryan79/PileOMeth). Sequencing data that did not meet these criteria were excluded from the study.

The data extracted using PileOMeth was taken forward for statistical analysis using IBM SPSS Statistics 24 and GraphPad prism 7. To correct for non-normal distribution, each methylation value at CpG sites in each individual were ranked with rank-based inverse normal transformations (Blom normal scores in SPSS). Unpaired multiple *t* tests were conducted on the ranked normal scores at each sequenced CpG site between cases vs controls for each gene separately. For multiple comparison testing, the false discovery rate approach was adopted. Discovery was determined using the two-stage linear step-up procedure of Benjamini, Krieger and Yekutieli, with *Q =* 10%. CpG sites with significant *P* values and *Q* values were then manually checked to ensure only sites with a noticeably visual difference in methylation between cases, and controls were included to investigate further. The effects of smoking and sex were assessed in our original data by excluding all non-smokers and females respectively, where we then reported *P* values. Logistic regression was performed using SPSS at each significant CpG site to adjust for age differences between cases vs controls. Age-adjusted odds ratios were reported for each CpG unless significance was lost.

### RNA extraction, genomic DNA removal and cDNA synthesis

VSMCs from the same samples as used in the NGS assay (*n* = 44) were used for gene expression analysis. RNA isolation was performed following the manufacturer’s protocol (QIAGEN RNeasy Mini Kit). Total RNA was eluted in 30 μl RNase-free water and taken forward for contaminating genomic DNA digestion using the manufacturer’s standard protocol (DNase I - RNase-Free: New England BioLabs M0303). CDNA was then synthesised from the DNase digested RNA using the ThermoFisher High-Capacity cDNA Reverse Transcription Kit (4368814) according to the manufacturer’s protocol. CDNA was eluted in a final volume of 40 μl and stored at − 20 °C until further use.

### TaqMan gene expression analysis

For each gene where differential methylation was observed after VSMC bisulphite sequencing (*SMYD2*, *IL6R*, *SERPINB9* and *ERG*), pre-designed TaqMan gene expression assays were purchased from Fisher Scientific UK. *GAPDH* was used as the normalisation reference gene:

- Hs01554629_m1 (*ERG* gene FAM-MGB dye)
- Hs00220210_m1 (*SYMD2* gene: FAM-MGB dye)
- Hs01075666_m1 (*IL6R* gene: FAM-MGB dye)
- Hs00394497_m1 (*SERPINB9* gene: FAM-MGB dye)
- Hs02758991_g1 (*GAPDH* gene: FAM-MGB dye)

The TaqMan qPCR assays were all performed on an Applied Biosystems Step One Plus qPCR system in duplicate and the qPCR constituents were as follows in a 20 μl total volume: 10 μl 2× TaqMan Gene Expression Master Mix (ThermoFisher), 1 μl 20× TaqMan Gene Expression primers/probes (ThermoFisher), 8 μl sterilised distilled water and 1 μl cDNA (25 ng/μl).

The TaqMan qPCR cycling conditions were as follows:

- 50 °C for 2 min – 1 cycle
- 95 °C for 20 s – 1 cycle
- 95 °C for 1 s – 40 cycles
- 60 °C for 20 s – 40 cycles

Mean Ct values of the target gene were subtracted from the mean Ct values of the housekeeper gene to acquire inverse delta Ct values (higher value represents higher expression).

### Immunohistochemistry to assess protein expression in aortic tissues

Histological staining of the Smyd2 protein was conducted on frozen sections from aortic tissues to corroborate differential *SMYD2* gene expression and promoter methylation in three AAAs and three controls. This work was conducted in the Histology Facility, Core Biotechnology Services, University of Leicester.

Three assays were conducted for each individual aortic tissue sample, meaning a total of 18 slides were prepared for microscopy. Six slides (three controls and three AAAs) were prepared with a Smyd2 polyclonal rabbit antibody at 50: 1 (ThermoFisher - PA5-51339), six control slides (three controls and three AAAs) were prepared with no primary antibody, and six slides (three controls and three AAAs) were prepared with a smooth muscle actin (SMA) polyclonal rabbit antibody at 100: 1 (ThermoFisher - PA5-19465). Visualisation of Smyd2 staining was concentrated on the regions where the highest density of smooth muscle fibres were seen from the SMA. The primary antibodies were detected using the NOVOLINK polymer detection system (Novocastra RE7140-K) using the standard protocol, and visualisation of the slides was performed on the Hamamatsu Nanozoomer 2.0HT Slide Scanner with the use of NDP VIEW2 software (U12388-01).

### Summary of statistical analysis

Statistical analyses were conducted using GraphPad Prism 7 (GraphPad Software, Inc., CA, USA) and IBM SPSS Statistics 24 (IBM, NY, USA). Continuous parametric data are presented as mean value ± standard error (SE) and non-parametric data are presented as median value and range; categorical data are presented as absolute value or percentage. The chi-square test was used to compare categorical data. Student’s *t* test was used to compare continuous parametric data. Pearson’s correlation coefficient was calculated to assess linear dependence between two variables. A *P* value of < 0.05 was considered statistically significant. Where applicable, a *Q* value has been reported (*Q* < 0.1), adjusted for false discovery rate (FDR).

## Additional file

Additional file 1: Supplemental material. (DOCX 592 kb)

## Abbreviations

AAA: Abdominal aortic aneurysm
ELISA: Enzyme-linked immunosorbent assay
HCY: Homocysteine
MeQTL: Methylation quantitative trait loci
NGS: Next-generation sequencing
PBMC: Peripheral blood mononuclear cell
UKAGS: UK aneurysm growth study
VSMC: Vascular smooth muscle cell
