# Supplementary material for: SMYD2 promoter DNA methylation is associated with abdominal aortic aneurysm (AAA) and SMYD2 expression in vascular smooth muscle cells
Source: Clin Epigenetics. 2018 Mar 2;10:29. doi: 10.1186/s13148-018-0460-9 (PMC5833080; doi:10.1186/s13148-018-0460-9)
Supplement: Supplementary file 1 — Supplemental material. (DOCX 592 kb) [file 13148_2018_460_MOESM1_ESM.docx]

# SUPPLEMENTAL MATERIAL

***SMYD2* promoter DNA methylation is associated with abdominal aortic aneurysm (AAA) and *SMYD2* expression in vascular smooth muscle cells**

**Authors**

Bradley J Toghill [a]*, Athanasios Saratzis [a], Peter J Freeman [b], Nicolas Sylvius [b], UKAGS collaborators, Matthew J Bown [a].

[a] Department of Cardiovascular Sciences and the NIHR Leicester Biomedical Research Centre, University of Leicester, LE27LX, UK.

[b] Department of Genetics, University of Leicester, LE17RH, UK.

* Corresponding author at: Department of Cardiovascular Sciences, Robert Kilpatrick clinical sciences building, Leicester Royal Infirmary, University of Leicester, LE27LX.

Email address: bt96@le.ac.uk (BJ Toghill)

## List of UKAGS collaborators:

Mr Rajiv Pathak: [rajiv.pathak@dgh.nhs.uk](mailto:rajiv.pathak@dgh.nhs.uk)

Mr Marcus J Brooks: [marcus.brooks@uhbristol.nhs.uk](mailto:marcus.brooks@uhbristol.nhs.uk)

Mr Paul Hayes: [paul.hayes@addenbrookes.nhs.uk](mailto:paul.hayes@addenbrookes.nhs.uk)

Prof Chris H Imray: [Christopher.Imray@uhcw.nhs.uk](mailto:Christopher.Imray@uhcw.nhs.uk)

Mr John Quarmby: [John.Quarmby@derbyhospitals.nhs.uk](mailto:John.Quarmby@derbyhospitals.nhs.uk)

Mr Sohail A Choksy: [sohailchoksy@nhs.net](mailto:sohailchoksy@nhs.net)

Mr Jonothon J Earnshaw: [Jonothan.Earnshaw@glos.nhs.uk](mailto:Jonothan.Earnshaw@glos.nhs.uk)

Prof Cliff P Shearman: [C.P.Shearman@soton.ac.uk](mailto:C.P.Shearman@soton.ac.uk)

Mr Eric Grocott: [eric.grocott@googlemail.com](mailto:eric.grocott@googlemail.com)

Mr Thomas Rix: [thomasrix@nhs.net](mailto:thomasrix@nhs.net)

Prof Ian C Chetter: [Ian.chetter@hey.nhs.uk](mailto:Ian.chetter@hey.nhs.uk)

Mr William Tennant: [william.tennant@nuh.nhs.uk](mailto:william.tennant@nuh.nhs.uk)

Mr Gabor Libertiny: [gabor.libertiny@ngh.nhs.uk](mailto:gabor.libertiny@ngh.nhs.uk)

Mr Tim Sykes: [Tim.Sykes@sath.nhs.uk](mailto:Tim.Sykes@sath.nhs.uk)

Mr Mark Dayer: [mark.dayer@tst.nhs.uk](mailto:mark.dayer@tst.nhs.uk)

Ms Lynda Pike: [lynda.pike@nhs.net](mailto:lynda.pike@nhs.net)

Mr Arun Pherwani: [Arun.Pherwani@uhns.nhs.uk](mailto:Arun.Pherwani@uhns.nhs.uk)

Mr Colin Nice: [colin.nice@ghnt.nhs.uk](mailto:colin.nice@ghnt.nhs.uk)

Mr Neil Browning: [neil.browning@asph.nhs.uk](mailto:neil.browning@asph.nhs.uk)

Prof Charles N McCollum: [charles.mccollum@manchester.ac.uk](mailto:charles.mccollum@manchester.ac.uk)

Mr Syed W Yusuf: [Syed.Yusuf@bsuh.nhs.uk](mailto:Syed.Yusuf@bsuh.nhs.uk)

Mr Mark Gannon: [mark.gannon@heartofengland.nhs.uk](mailto:mark.gannon@heartofengland.nhs.uk)

Mr Jamie Barwell: [Jamie.Barwell@nhs.net](mailto:Jamie.Barwell@nhs.net)

Mrs Sara Baker: [Sara.Baker@rbch.nhs.uk](mailto:Sara.Baker@rbch.nhs.uk)

Mr Srinivasa R Vallabhaneni: [fempop@liverpool.ac.uk](mailto:fempop@liverpool.ac.uk)

Mr JV Smyth: [JV.Smyth@cmft.nhs.uk](mailto:JV.Smyth@cmft.nhs.uk)

Prof Alun H Davies: [a.h.davies@imperial.ac.uk](mailto:a.h.davies@imperial.ac.uk)

Mr Tim Lees: [Tim.Lees@nuth.nhs.uk](mailto:Tim.Lees@nuth.nhs.uk)

Mr Louis Fligelstone: [Louis.Fligelstone@wales.nhs.uk](mailto:Louis.Fligelstone@wales.nhs.uk)

Prof Rob Sayers: [rs152@leicester.ac.uk](mailto:rs152@leicester.ac.uk)

Prof Nilesh J Samani: [njs@leicester.ac.uk](mailto:njs@leicester.ac.uk)

Dr Mike J Sweeting: [mjs212@medschl.cam.ac.uk](mailto:mjs212@medschl.cam.ac.uk)

Prof John Thompson: [trj@le.ac.uk](mailto:trj@le.ac.uk)

## Supplemental Figures


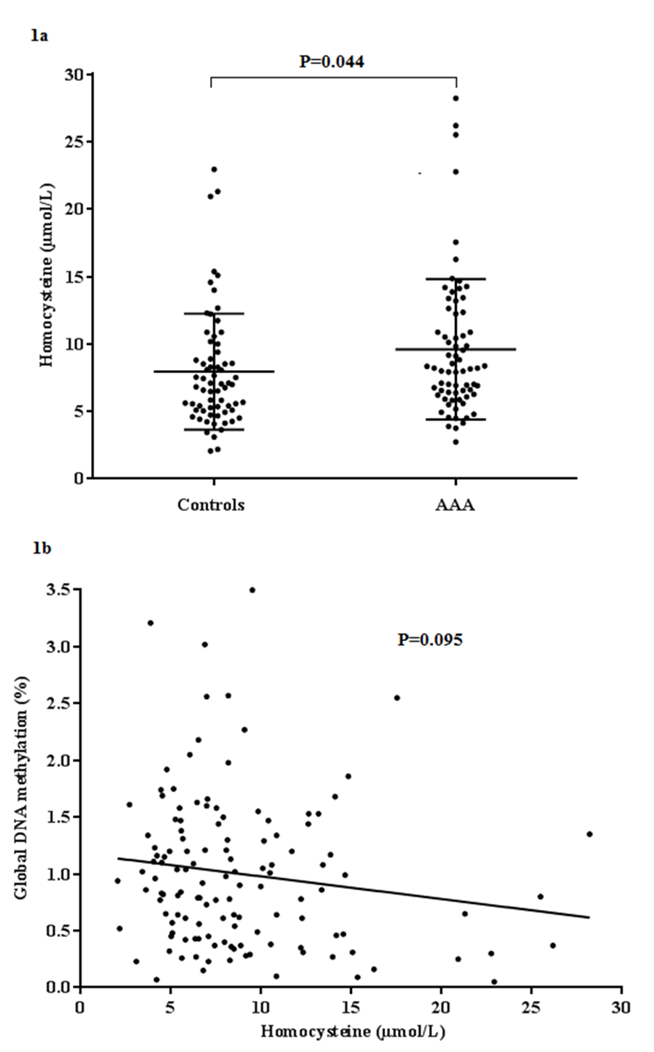


**Supplemental Figure 1a:** Circulating homocysteine levels in matched (from global methylation assay) blood plasma of controls (n=67) and AAA (n=70). Plasma homocysteine levels were higher in men with AAA compared to controls (9.6 µmol/L ± 0.62, n=70, vs 7.94 µmol/L ± 0.52, n=67, p=0.0433). **Figure 1b**: The linear relationship between global methylation % and circulating homocysteine (n=129), where there was no significant association (P=0.095).


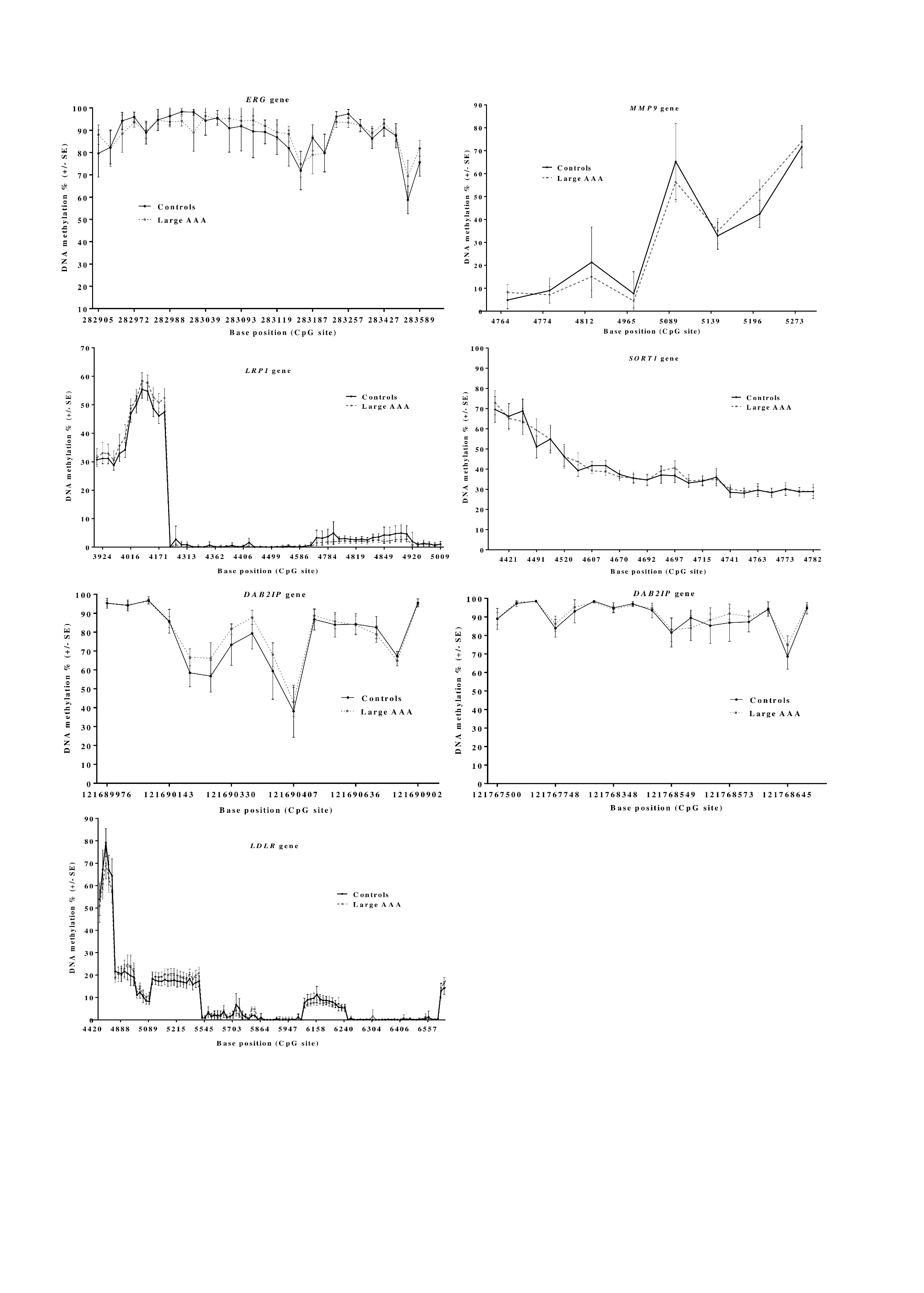


**Supplemental Figure 2:** Vascular smooth muscle cell DNA methylation status of bisulphite sequenced CpG islands where no significant differential methylation was observed in 20 controls vs 24 AAA (*ERG, MMP9, LRP1, SORT1*, *DAB2IP* and *LDLR*).


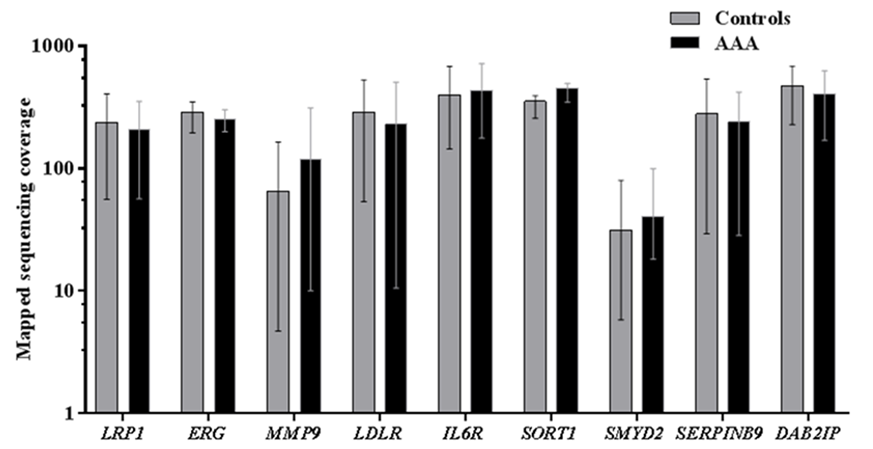


**Supplemental Figure 3:** Mean coverage of mapped sequencing reads in candidate gene CpG Islands. Analysis performed on VSMC DNA (24 AAA and 20 controls). Error bars represent standard deviation.

## Supplemental Tables

|  | 48 Large AAA  (>55mm) | 45 Small AAA  (30-55mm) | 92 Controls  (<25mm) |
| --- | --- | --- | --- |
| Sex | Male | Male | Male |
| Ethnicity | Caucasian | Caucasian | Caucasian |
| Smoking | Over 10 years | Over 10 years | Over 10 years |
| Median age (range) | 74 (30) | 70 (18) | 68 (34) |
| Mean aortic diameter (mm (+/- SD)) | 68 (0.96) | 44 (0.68) | 19 (0.1) |

**Supplemental Table 1:** Demographic summary of samples used for peripheral blood global DNA methylation analysis.

|  | 24 Large AAA  (>55mm) | 20 Cadaveric controls  (<25mm) | |
| --- | --- | --- | --- |
| No of males | 24 | 16 |  |
| No of females | 0 | 4 |  |
| Ethnicity | Caucasian | Caucasian |  |
| Smokers | 22 | 16 |  |
| Median age (range) | 68 (25) | 56 (35) |  |
| Mean aortic diameter (mm (+/- SD)) | 65 (1.2) | Exact values unknown |  |
|  |  |  |  |

**Supplemental Table 2:** Demographic summary of samples where vascular smooth muscle cell DNA was bisulphite treated and used for next generation sequencing.

|  | 70 AAA  (30-71mm) | 67 Controls  (<25mm) |
| --- | --- | --- |
| Sex | Male | Male |
| Ethnicity | Caucasian | Caucasian |
| Smoking | Over 10 years | Over 10 years |
| Median age (range) | 73 (28) | 68 (34) |
| Mean aortic diameter (mm (+/- SD)) | 51 (1.2) | 19 (0.1) |

**Supplemental Table 3:** Demographic summary of samples used for homocysteine analysis in blood plasma.
